# Robotic-Assisted Thoracic Surgery in the Immunotherapy Era: Navigating Altered Anatomy, Oncologic Precision, and the Future of Integrated Platforms

**DOI:** 10.3390/jcm15124485

**Published:** 2026-06-10

**Authors:** Dimitrios E. Magouliotis, Vasiliki Androutsopoulou, Ugo Cioffi, Vanesa Brecher, Andrew Xanthopoulos, Fabrizio Minervini, Marco Scarci

**Affiliations:** 1 Department of Cardiac Surgery Research, Lankenau Institute for Medical Research, Wynnewood, PA 19096, USA; vb4075@pcom.edu; 2 Department of Cardiothoracic Surgery, University of Thessaly, Biopolis, 41110 Larissa, Greece; androutsopoulouvasiliki@uth.gr; 3 Department of Surgery, University of Milan, 20157 Milan, Italy; ugo.cioffi@guest.unimi.it; 4 Department of Cardiology, University of Thessaly, Biopolis, 41110 Larissa, Greece; andrewvxanth@gmail.com; 5 Department of Thoracic Surgery, Luzern Kanton Hospital, 6000 Luzern, Switzerland; fabriziominervini@hotmail.com; 6 Department of Cardiothoracic Surgery, Hammersmith Hospital, Imperial College Healthcare, National Health Service (NHS) Trust, London W2 1NY, UK; marco.scarci@nhs.net

**Keywords:** robotic-assisted thoracic surgery, neoadjuvant immunotherapy, non-small cell lung cancer, RATS, immune checkpoint inhibitors, lung resection

## Abstract

The adoption of neoadjuvant immune checkpoint inhibitor (ICI)-based chemoimmunotherapy has fundamentally transformed the operative landscape of resectable non-small cell lung cancer (NSCLC). Surgeons are now routinely confronted with ICI-altered tissue planes characterized by hilar fibrosis, vascular friability, and disrupted lymph node architecture. Simultaneously, robotic-assisted thoracic surgery (RATS) has consolidated its position as the dominant minimally invasive platform for pulmonary resection, accounting for the majority of lobectomies and segmentectomies performed at high-volume centers in 2023. Whether RATS confers specific technical advantages in this increasingly complex operative context remains incompletely characterized. We conducted a structured narrative review of published evidence, synthesizing data from randomized controlled trials, prospective cohorts, national registry analyses, and emerging technology reports addressing RATS in the setting of neoadjuvant ICI-based therapy for NSCLC. A systematic literature search was conducted across PubMed and EMBASE using predefined search terms. Available evidence, though largely retrospective and limited by small sample sizes, consistently demonstrates that RATS after neoadjuvant chemoimmunotherapy is technically feasible and oncologically sound, with R0 resection achievable in virtually all cases. The enhanced three-dimensional visualization, tremor filtration, and instrument degrees of freedom afforded by robotic platforms appear particularly advantageous in the setting of dense hilar adhesions and fragile pulmonary vasculature. Lymph node yield, a recognized robotic advantage, is preserved or enhanced despite post-ICI fibrosis. Pooled conversion rates to thoracotomy, derived from post hoc surgical analyses of ICI trial populations rather than trials designed to measure conversion, are higher than for upfront resection; available retrospective single-center data, including one direct RATS-versus-VATS comparison, suggest lower conversion rates with RATS in experienced hands, though this conclusion requires prospective validation. Emerging platform integrations, including combined robotic bronchoscopy and thoracoscopic surgery, single-port systems, and artificial intelligence-assisted anatomical navigation, are poised to further extend the reach of minimally invasive surgery in this challenging clinical scenario. In experienced centers, RATS appears to offer a technically favorable minimally invasive platform for pulmonary resection after neoadjuvant ICI-based therapy, with potential advantages over VATS in managing immunotherapy-altered anatomy; however, this conclusion is derived from retrospective series and should be interpreted cautiously pending prospective comparative data. Prospective multicenter trials with standardized surgical endpoints are urgently needed.

## 1. Introduction

Non-small cell lung cancer (NSCLC) remains the leading cause of cancer-related mortality worldwide, with surgical resection constituting the cornerstone of curative-intent management for localized and locally advanced disease [1]. Over the past decade, the treatment paradigm for resectable and potentially resectable NSCLC has undergone a fundamental transformation, driven by the integration of immune checkpoint inhibitors (ICIs) into perioperative protocols. Landmark trials including CheckMate 816 [2], NADIM [3], and the KEYNOTE-671 [4] platform have firmly established neoadjuvant chemoimmunotherapy as a standard of care for stage IB–IIIA NSCLC, demonstrating superior rates of major pathological response (MPR) and pathological complete response (pCR) compared with chemotherapy alone, translating into meaningful improvements in event-free survival.

Simultaneously, robotic-assisted thoracic surgery (RATS) has undergone its own quiet revolution. From a niche innovation confined to a handful of specialized centers in the early 2000s, RATS has emerged as the dominant platform for minimally invasive pulmonary resection in high-volume programs. According to the 2025 Society of Thoracic Surgeons (STS) Database report, robotic surgery accounted for the majority of lobectomies and segmentectomies performed among database participants in 2023, surpassing video-assisted thoracoscopic surgery (VATS) for the first time [5]. This transition reflects the recognized technical advantages of robotic platforms: superior three-dimensional (3D) visualization, tremor filtration, enhanced instrument degrees of freedom (up to seven axes of motion), and ergonomic benefits for the operating surgeon.

The convergence of these two trends creates a clinically urgent and underappreciated problem. Surgeons operating in the post-ICI era are no longer encountering virgin anatomy. Immune activation, inflammatory cascades, and the tissue-remodeling consequences of checkpoint inhibition produce a distinct intraoperative phenotype: dense hilar adhesions, fused fissures, calcified and inflamed lymph nodes, and friable pulmonary vasculature. These changes render even expert-level thoracoscopic dissection considerably more challenging. The pooled conversion rate to open thoracotomy following neoadjuvant chemoimmunotherapy has been reported at approximately 20% in post hoc surgical analyses of prospective ICI trials [6]—a figure that, despite heterogeneous conversion definitions and mixed surgical approaches across included studies, underscores the magnitude of the operative challenge and raises the question of whether specific surgical platforms are better suited to this environment.

Despite the clinical urgency of this question, the literature remains fragmented, dominated by small single-center retrospective series and post hoc analyses of trial populations in which surgical approach was not standardized. No comprehensive synthesis has yet examined the intersection of RATS and ICI-based neoadjuvant therapy as a primary focus. The present review addresses this gap. Following a structured literature search (detailed in Section 2), we narratively examine the biological substrate of immunotherapy-altered anatomy, synthesize the available clinical evidence for RATS in the post-ICI context, describe technical adaptations that optimize robotic performance in this setting, and outline emerging platform integrations (such as combined robotic bronchoscopy and thoracoscopy, single-port RATS, and artificial intelligence (AI)-assisted anatomical navigation) that are poised to define the next generation of thoracic oncologic surgery.

## 2. Methods

This narrative review was conducted in accordance with guidance for structured narrative reviews in the surgical sciences. A systematic literature search was performed in PubMed and EMBASE from inception through April 2026. The following Medical Subject Headings (MeSH) terms and keywords were used in combination: “robotic-assisted thoracic surgery”, “RATS”, “neoadjuvant immunotherapy”, “immune checkpoint inhibitor”, “neoadjuvant chemoimmunotherapy”, “non-small cell lung cancer”, “NSCLC”, “pulmonary resection”, “lobectomy”, “segmentectomy”, “conversion to thoracotomy”, and “lymph node dissection”. Supplementary searches were conducted for specific technology domains, including “robotic bronchoscopy”, “uniportal RATS”, and “artificial intelligence thoracic surgery”. No language restrictions were applied. Inclusion criteria encompassed original research articles, systematic reviews and meta-analyses, prospective and retrospective cohort studies, and technology reports addressing RATS or minimally invasive surgery in the context of neoadjuvant ICI-based therapy for NSCLC. Case reports with fewer than five patients and conference abstracts without full-text publication were excluded. Study selection was performed by two independent reviewers (D.E.M. and V.A.), with discrepancies resolved by consensus. Given the heterogeneity of included studies in terms of design, surgical approaches, and reported outcomes, formal meta-analysis was not performed; findings are presented as a narrative synthesis with explicit acknowledgment of study quality and evidence level where relevant.

## 3. Immunotherapy-Altered Tissue Biology: The Surgeon’s New Operative Reality

### 3.1. ICI-Induced Hilar Fibrosis and Adhesion Formation: Pathophysiological Basis

To understand why surgery after ICI-based therapy is fundamentally different, one must first understand the biology of immune activation in the mediastinum and hilum. Programmed death-1 (PD-1) and programmed death ligand-1 (PD-L1) checkpoint inhibitors work by releasing T-cell suppression, unleashing a broad antitumor immune response that is not anatomically restricted to the tumor microenvironment. In the chest, this systemic immune activation can trigger a paratumoral and perihilar inflammatory reaction characterized by macrophage infiltration, cytokine elaboration, and secondary fibroblast activation [7]. The pathological consequence, observable intraoperatively, is a dense fibrinous or fibrotic reaction within the hilum, mediastinal fat, and along the pleural surfaces, regardless of whether the primary tumor itself achieves a deep pathological response.

This phenomenon has been recognized in pathological studies of surgical specimens from patients who received neoadjuvant ICI therapy. In the CheckMate 816 trial, intraoperative findings reported by surgeons included hilar inflammation and fibrosis in a substantial proportion of cases, contributing to extended operative times and higher rates of technical difficulty [2]. Bott and colleagues documented that 7 of 13 minimally invasive procedures in their early series of resections after nivolumab required conversion to thoracotomy, attributing this predominantly to ICI-related hilar inflammation and fibrosis [8]. The mechanistic driver appears to be transforming growth factor-beta (TGF-β)-mediated fibroblast activation, potentiated by the robust interferon-gamma (IFN-γ) signaling associated with effective T-cell engagement [9]. Importantly, the severity of fibrotic response does not appear to correlate linearly with depth of pathological tumor response. As suggested by emerging evidence, a patient with a pCR may paradoxically present with more florid perihilar fibrosis than one with residual viable tumor, as the former reflects a more vigorous immune reaction [10,11,12,13]; this observation warrants further prospective validation.

### 3.2. Impact on Tissue Planes, Vascular Friability, and Lymph Node Architecture

**Figure 1:**
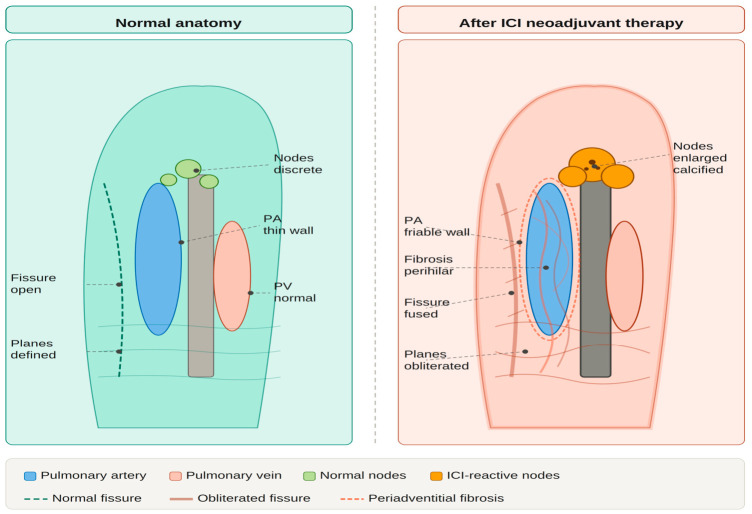
Normal (**left**) versus ICI-altered (**right**) hilar anatomy. The grey central structure represents the bronchus. Dashed green lines denote the normal, patent interlobar fissure with preserved dissection planes; solid brown lines denote the obliterated, fibrotic fissure with planes lost. The dashed orange outline indicates periadventitial fibrosis encasing the pulmonary artery, which renders its wall friable. ICI: Immune Checkpoint Inhibitor; PA: Pulmonary Artery; PV: Pulmonary Vein.

The practical intraoperative consequences of ICI-mediated tissue remodeling manifest across three anatomical domains that collectively define the operative challenge of post-ICI thoracic surgery. First, tissue planes become obliterated or densely adherent. The interlobar fissures, which provide the primary anatomical guide for lobe-specific dissection, may be completely fused, rendering the conventional fissure-first approach frequently untenable and risking inadvertent entry into vascular structures or airway walls during attempted plane development [10]. Second, the pulmonary vasculature, particularly the pulmonary artery and its lobar branches, may exhibit wall changes secondary to periadventitial inflammation and fibrosis, rendering vessels more prone to tearing during retraction or clamp application, with stapling across inflamed tissue carrying an increased risk of dehiscence or hemorrhage [11]. Third, mediastinal and hilar lymph nodes undergo a distinct immunological transformation: ICI therapy induces T-cell infiltration, germinal center formation, and reactive lymph node enlargement, with calcification occurring preferentially in previously involved nodes that have responded to therapy [12]. These changes convert lymphadenectomy from a relatively straightforward capsular dissection into a meticulous, node-by-node removal from inflamed and adherent surrounding structures. The contrast between normal and ICI-altered hilar anatomy is illustrated schematically in Figure 1, which depicts the key pathological features defining the post-ICI operative environment, including obliterated fissures, periadventitial fibrosis enveloping the pulmonary artery, and enlarged calcified reactive lymph nodes.

### 3.3. The Pathological Complete Response Paradox

One of the most counterintuitive observations in the post-ICI surgical literature is what we term the pCR paradox: the patients who derive the greatest oncological benefit from neoadjuvant ICI therapy are those achieving a pathological complete response; the same patients may paradoxically present the greatest intraoperative technical challenges. This occurs because pCR reflects the most robust and sustained immune activation, which in turn generates the most intense secondary inflammatory-fibrotic response in the surgical field [13]. In the NADIM trial, which reported pCR rates of 71% with nivolumab plus chemotherapy in stage III NSCLC, surgeons documented extensive fibrosis and adhesion formation at the time of resection as among the most technically demanding in their operative experience [3]. Pathologists examining surgical specimens from pCR patients often document extensive fibrohyaline replacement of the original tumor bed, surrounded by a dense inflammatory infiltrate extending into the mediastinal fat.

This paradox has important implications for operative planning. Preoperative imaging, while essential for restaging, cannot reliably predict the degree of intraoperative technical difficulty. A radiologically complete or near-complete response should not be interpreted as a signal that surgery will be straightforward; instead, it is quite the opposite. Surgeons must be prepared for the most demanding dissection precisely in the patients with the best oncologic outcomes.

### 3.4. Radiologic Correlates: CT Predictors of Intraoperative Difficulty After ICI

Several computed tomography (CT) findings at restaging have been proposed as surrogate markers of intraoperative complexity after neoadjuvant ICI therapy. Ground-glass opacity surrounding the primary tumor, new or increased pleural thickening, perihilar haziness, and mediastinal fat stranding are each associated with an increased likelihood of dense intraoperative adhesions [14]. Enlarged mediastinal nodes that fail to decrease in size despite radiologic tumor response, the so-called discordant nodal response, frequently represent reactive immunological enlargement rather than residual metastatic disease, and often prove the most technically challenging structures to dissect [15]. Positron emission tomography (PET) findings showing persistent or new hilar metabolic activity in the absence of viable tumor may similarly flag the expected intraoperative environment. Integrating these radiologic signals into preoperative planning conversations with anesthesia, nursing, and the bedside surgical team can facilitate appropriate preparation for extended operative times, availability of vascular surgery backup, and a low threshold for conversion.

## 4. RATS After Neoadjuvant Chemoimmunotherapy: Current Evidence

### 4.1. Overview of the Evidence Base

The evidence base for RATS specifically in the post-ICI neoadjuvant context remains limited in volume but is rapidly expanding. The available literature comprises a handful of dedicated single-institution retrospective studies, post hoc surgical subgroup analyses from prospective ICI trials, and the first systematic reviews and meta-analyses addressing the feasibility and safety of minimally invasive surgery after chemoimmunotherapy [6] (Table 1). A critical interpretive challenge is that most prospective neoadjuvant ICI trials did not mandate surgical approach or prospectively collect granular intraoperative data; surgical details are therefore often sparse or absent in the primary trial reports. The landmark randomized controlled trials comparing RATS and VATS (including ROMAN, RAVAL-4, and the study by Lamas et al. published in 2026) focused on upfront resection populations and did not systematically enroll post-ICI patients [16]. Extrapolation to the post-ICI context, while informative, must therefore be made with appropriate caution.

### 4.2. Conversion Rates: RATS vs. VATS in the Post-ICI Setting

Conversion to open thoracotomy is the sentinel intraoperative outcome in the post-ICI surgical literature, reflecting the aggregate technical difficulty of operating in immunotherapy-altered anatomy. The systematic review and meta-analysis by Bertoglio and colleagues, published in 2026, pooled data from 27 prospective trials involving 2691 patients with resectable NSCLC treated with ICI-based regimens before surgery [6]. Minimally invasive surgery was employed in 47% of resections, with a pooled conversion rate of 20%. It is important to note that this figure derives from post hoc surgical analyses of ICI trials not designed to prospectively capture conversion rates, and reflects a heterogeneous literature with variable conversion definitions and mixed surgical approaches. Nonetheless, it is substantially higher than conversion rates reported for RATS in upfront resection cohorts, which typically range from 2% to 7% in experienced centers [17].

Direct head-to-head comparisons between RATS and VATS in the post-ICI context are available from a small number of institutional series. At Thomas Jefferson University, a high-volume robotic thoracic center, Mack and colleagues retrospectively compared 21 patients who underwent robotic lobectomy after neoadjuvant chemoimmunotherapy (Checkmate-816 protocol) against 64 patients undergoing upfront robotic resection [18]. No conversions to thoracotomy occurred in either group, and R0 resection was achieved in all cases, though operative time was significantly longer in the post-ICI cohort (224 vs. 177 min, *p* = 0.011). The lymph node yield was higher in the post-ICI group (median 21 vs. 16.5 nodes, *p* = 0.042), a finding that likely reflects the immunological enlargement and accessibility of reactive nodes.

Comparative data from Shanghai Chest Hospital, analyzing RATS vs. VATS lobectomy following neoadjuvant immunochemotherapy in NSCLC patients, demonstrated that RATS was associated with reduced surgical-related ICU stay, superior N1 lymph node station assessment, and similar recurrence-free survival profiles [19]. In a multicenter Italian series comparing robotic surgery to open thoracotomy after neoadjuvant treatment for locally advanced NSCLC, robotic surgery demonstrated favorable perioperative outcomes including shorter hospital stay and lower complication rates, despite thoracotomy remaining the comparator standard at many centers [20]. The convergent message across these retrospective series, conducted at institutions with high-volume robotic programs, is that RATS is technically feasible after ICI therapy and, in the limited comparative data available, appears to be associated with lower conversion rates than VATS in experienced hands. Critically, however, only the Pan et al. series included a direct RATS-versus-VATS comparator group in a post-ICI population [19]; the other studies compared RATS against upfront RATS or open thoracotomy. These conclusions should therefore be interpreted cautiously, as they may not generalize to lower-volume centers or surgeons earlier on the robotic learning curve. The technical advantages of RATS in this context are most fully realized in high-volume programs with established robotic thoracic surgery infrastructure, and their applicability to institutions without such experience requires prospective study.

### 4.3. Lymph Node Yield: The Robotic Advantage in the Fibrotic Hilum

Adequate mediastinal lymph node dissection is both an oncological imperative and a prognostic determinant in NSCLC surgery [21]. ICI-induced lymph node changes, such as reactive enlargement, capsular fibrosis, and pericapsular adhesions, transform what is routinely a systematic anatomical dissection into a demanding exercise in tissue identification and careful hemostasis. The robotic platform offers theoretical advantages in this context: tremor-free instrument tips, magnified 3D visualization that reveals tissue plane boundaries invisible to the naked eye, and wristed instruments capable of precisely navigating the narrow corridors between inflamed nodes and adjacent vascular structures.

The empirical data are supportive. Across published robotic series in the post-ICI context, lymph node yields are consistently at or above the thresholds recommended by oncologic guidelines [20,22]. The Jefferson series reported a median of 21 lymph nodes in the post-ICI RATS cohort [18], exceeding the STS-recommended threshold and comparing favorably with VATS series in similar populations. This finding carries oncological significance: adequate nodal staging not only guides adjuvant therapy decisions but also influences survival independently of T-stage [21]. The robotic advantage in lymph node yield appears to be preserved or even amplified in the post-ICI setting. The technical demands of lymphadenectomy in post-ICI patients, however, deserve specific elaboration. Calcified, inflamed, and pericapsularly adherent nodes pose risks distinct from those encountered in untreated anatomy: attempted en bloc removal may result in inadvertent entry into the pulmonary artery or superior vena cava, and traction-related dehiscence of calcified capsular planes can trigger hemorrhage that is difficult to control thoracoscopically. The recommended approach is a systematic node-by-node capsular dissection, proceeding from the most accessible station to the most adherent, using cold sharp robotic scissors to develop the capsular plane rather than energy devices in proximity to vascular structures. Robotic magnification at 10x or greater allows identification of the fine tissue plane between the nodal capsule and the adventitia of adjacent vessels, a plane that is effectively invisible at standard thoracoscopic magnification. When calcification has bridged the plane between a node and the bronchial wall or vascular adventitia, limited sharp excision of the adherent tissue with frozen section examination of the margin is preferable to forcible avulsion. A low threshold for placing hemostatic clips on small perihilar vessels before they are transected reduces the risk of obscuring hemorrhage in a tight, inflamed field.

**Table 1:** Summary of published studies reporting outcomes of robotic-assisted thoracic surgery (RATS) after neoadjuvant immune checkpoint inhibitor (ICI)-based therapy for non-small cell lung cancer.

Study (Year)	N	Stage	Regimen	Approach	Conv. (%)	R0 (%)	Nodes (med.)	LOS (d)
Gao et al. (2022) [23]	44	IIIA–IIIB	Chemo + ICI × 3	RATS	0	100	NR	NR
Mack et al. (2024) [18]	21	I–IIIA	Nivolumab + chemo (CM-816)	RATS	0	100	21	2
Pan et al. (2023) [19]	NR	I–IIIA	Chemo-immunotherapy	RATS vs. VATS	Lower in RATS	Similar	Higher in RATS	Shorter in RATS
Gallina et al. (2025) [10]	25	IIIA	Chemo ± ICI	RATS vs. Open	NR	NR	NR	Shorter in RATS
Bertoglio et al. (2026) [6]	2691	IB–IIIA	ICI-based (pooled)	MIS (47%)/Open	20 (pooled)	NR	NR	NR
Lamas et al. (2026) [16]	Pooled RCTs	I–III	Upfront (no ICI)	RATS vs. VATS	Similar	Similar	Higher in RATS	Similar

CM-816, CheckMate-816 protocol; Conv., conversion to open thoracotomy; d, days; ICI, immune checkpoint inhibitor; LOS, length of hospital stay; med., median; MIS, minimally invasive surgery; NR, not reported; RATS, robotic-assisted thoracic surgery; RCT, randomised controlled trial; VATS, video-assisted thoracoscopic surgery.

### 4.4. Perioperative Outcomes: Operative Time, Blood Loss, Length of Stay, and Complications

Beyond conversion rates and lymph node yield, the perioperative outcome profile of RATS after ICI therapy appears acceptable, albeit with predictable differences compared to upfront resection cohorts. Operative times are consistently prolonged, reflecting the technical burden of dissection in altered anatomy [18]. Estimated blood loss and transfusion rates do not appear to be substantially elevated in carefully selected patients at experienced centers [19]. Length of hospital stay (a sensitive surrogate for composite postoperative recovery) is preserved at levels comparable to upfront RATS when robotic conversion is avoided: the Jefferson series reported a median stay of 2 days in both cohorts [18]. Complication profiles, including air leak duration, atelectasis, pneumonia, and arrhythmia, are broadly consistent with published benchmarks, though direct comparisons with VATS or open surgery in matched post-ICI populations are not yet available.

Surgical delay, the interval between completion of neoadjuvant therapy and the date of surgery, was reported in approximately 9% of patients in the Bertoglio meta-analysis [6], often attributable to immune-related adverse events (irAEs) requiring steroid management. Interestingly, steroid-treated irAEs may paradoxically facilitate surgery by partially attenuating the fibrotic response, though this hypothesis requires prospective investigation. Pneumonectomy was required in approximately 10% of patients in the pooled population [6], reflecting cases where parenchymal-sparing resection proved technically impossible due to hilar involvement or inability to achieve negative margins short of whole-lung removal.

### 4.5. Oncologic Outcomes: R0 Resection, Recurrence-Free Survival, and Overall Survival

The ultimate measure of surgical adequacy in the post-ICI context is oncologic outcome. The available evidence, though limited in follow-up duration, is encouraging. R0 resection rates in dedicated RATS series after neoadjuvant ICI therapy approach 100% at experienced centers [18], a finding that speaks to the technical precision afforded by the robotic platform even in anatomically distorted operative fields. Major pathological response rates reported in surgical series following RATS and neoadjuvant chemoimmunotherapy range from approximately 65% to 82%, with pCR rates of 45–59% [23], consistent with the landmark randomized trial data and confirming that neoadjuvant treatment is exerting its intended effect.

Long-term survival data specifically for RATS after ICI therapy are not yet mature. Recurrence-free survival curves from the Shanghai series suggested outcomes comparable between RATS and VATS cohorts [19], while pooled analyses from neoadjuvant ICI trials, regardless of surgical approach, demonstrate meaningful improvements in event-free survival compared to historical chemotherapy-only benchmarks [4]. The inference that RATS does not compromise oncologic outcomes relative to VATS or open surgery in this context is supported by the totality of evidence, though direct comparative randomized data are lacking and urgently needed.

### 4.6. Stage III NSCLC: Where the Evidence Is Most Urgent

Stage III NSCLC represents the clinical scenario where the intersection of aggressive neoadjuvant ICI therapy and complex surgery is most consequential and most understudied. Patients with stage IIIA–IIIB disease who receive neoadjuvant chemoimmunotherapy present to the operating room with the largest primary tumors, the most extensively involved mediastinal nodal stations, and the greatest degree of ICI-mediated tissue remodeling [23]. Several prospective cohort studies have specifically examined RATS in this population. A real-world prospective study from China enrolled 44 patients with stage IIIA–IIIB NSCLC who received three cycles of neoadjuvant chemoimmunotherapy followed by RATS [23]. Major pathological response was achieved in 81.8% of patients, and pCR in 59.1%. Intraoperative findings consistently revealed adhesion and fibrosis, edema, and microbleeds in the chest, a characterization that precisely maps to the pathophysiological framework outlined in Section 2. Despite these challenges, surgical completion was achieved in all patients.

## 5. Technical Adaptations for RATS in Immunotherapy-Treated Patients

### 5.1. Preoperative Planning and Team Preparation

The technical execution of RATS after ICI therapy begins well before the patient enters the operating room. Meticulous preoperative planning must incorporate the radiologic findings discussed in Section 3.4, with explicit communication to the entire operative team (e.g., anesthesia, nursing, and the bedside assistant) regarding the anticipated degree of technical complexity. A structured pre-case “timeout” should assign specific roles for emergency scenarios: thoracotomy initiation, robot undocking, blood product access, and anesthesia escalation [24]. The critical importance of a highly skilled bedside assistant cannot be overstated in the post-ICI context. Because the console surgeon is physically distant from the operative field, the bedside assistant serves as the primary responder to any intraoperative crisis, and must be independently capable of managing the thoracoscopic field, applying direct pressure to vascular injuries, and initiating conversion without waiting for the console surgeon to scrub in [24].

**Figure 2:**
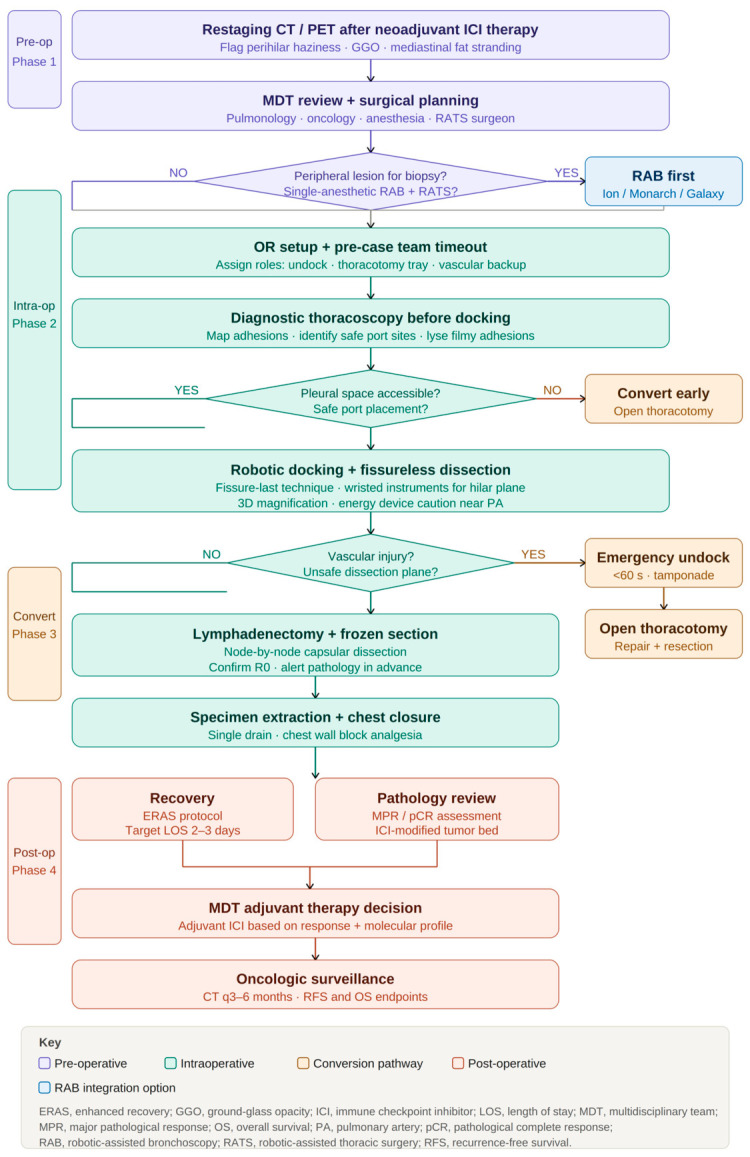
An integrated surgical decision pathway encompassing all phases of the perioperative journey. This pathway represents a proposed expert-consensus framework and has not been prospectively validated as a clinical guideline. Supporting references for each phase are provided in the relevant sections of the main text.

Single-lumen intubation with a bronchial blocker is preferred over double-lumen endotracheal intubation in cases planned for combined robotic bronchoscopy and thoracoscopy, with transition to double-lumen tube accomplished at the time of thoracoscopic port placement if needed. Epidural analgesia or paravertebral block placement prior to positioning reduces the likelihood that pain-related patient movement will compromise instrument trajectory during delicate hilar dissection. An integrated surgical decision pathway encompassing all phases of the perioperative journey, going from preoperative staging and MDT planning through intraoperative decision-making (Table 2), conversion protocols, and finally postoperative management, is presented in Figure 2.

### 5.2. Port Placement and Docking in Scarred or Adherent Chests

Standard port placement templates for robotic lobectomy may require modification in the post-ICI patient. Pleural adhesions, ranging from filmy to tenacious and vascularized, frequently prevent safe initial entry at conventional sites. A diagnostic thoracoscopy with the 0° camera prior to robotic docking is strongly recommended to map the adhesion pattern, identify safe windows for port insertion, and permit lysis of adhesions before the robot is docked [25]. In cases with total or near-total obliteration of the pleural space, an extrapleural approach or conversion to a staged procedure should be considered early rather than persisting with suboptimal access.

Once the pleural space is safely entered and the adhesion burden mapped, docking should be optimized for the expected axis of dissection. In right upper and middle lobe resections, where ICI-related fibrosis most commonly concentrates at the anterior hilum, an anterior docking angle with dedicated arm positioning for the superior mediastinum is preferred. For lower lobe resections, where the inferior pulmonary ligament and posterior hilum are key dissection territories, a posterior or lateral docking configuration provides better instrument triangulation [25].

### 5.3. Dissection Techniques for Fused Fissures and Calcified Nodes

Fissure dissection is among the most technically demanding components of post-ICI lobectomy. The “fissure-last” or “fissureless” technique, in which the pulmonary artery and bronchus are individually secured before the fissure is divided, is strongly preferred in the post-ICI context, as it avoids the need to develop the fissure as a primary step and reduces the risk of entering the fissural vascular plexus [26]. The robotic platform’s wristed instruments are particularly valuable here, permitting precise dissection along the arterial adventitia even in tight, inflamed spaces where straight VATS instruments would require exaggerated fulcrum maneuvers.

Lymph node dissection in the post-ICI patient should proceed systematically but with heightened attention to vascular anatomy. Reactive nodes are frequently adherent to the bronchial wall, the pulmonary artery, or the superior vena cava, and their removal requires deliberate capsular dissection rather than en bloc removal. Energy devices should be used judiciously in proximity to the pulmonary artery, where thermal spread to an already-friable vessel wall can be catastrophic [27]. Cold sharp dissection with robotic scissors, combined with hemostatic clips, is preferred near major vascular structures.

### 5.4. Vascular Control Strategies and Emergency Conversion Protocols

Vascular injury during RATS in the post-ICI setting is the most feared intraoperative complication, and its management requires advance preparation. The most common mechanism is inadvertent entry into an inflamed, friable pulmonary artery branch during fissural dissection or lymphadenectomy [28]. The robotic platform offers a potential advantage in this scenario compared to VATS: the console surgeon’s unimpeded view of the bleeding source, combined with the bedside assistant’s ability to apply direct digital pressure, provides a controlled interval during which the robot can be undocked and thoracotomy initiated. However, this advantage is predicated on a practiced team with clearly assigned roles and a rehearsed undocking sequence timed to under 60 s [24]. All post-ICI RATS cases should be performed with an open thoracotomy tray immediately available, and the skin incision should be marked prior to docking.

In cases where the primary surgeon anticipates exceptional vascular complexity (such as a tumor with direct pulmonary artery involvement in the setting of pCR-associated hilar fibrosis) consideration should be given to vascular surgical standby or conversion planning as a staged approach. Pneumonectomy, when required, should be performed with particular attention to bronchial stump coverage given the ICI-related impairment of local tissue healing capacity [29].

### 5.5. Role of Intraoperative Frozen Section and Pathologic Guidance

ICI therapy fundamentally alters the gross and histological appearance of the resected specimen, making intraoperative assessment of margin adequacy more challenging [30]. In cases of pCR, the tumor bed is replaced by fibrohyaline scar tissue, which may be grossly indistinguishable from the surrounding parenchyma. Intraoperative frozen section of bronchial and vascular margins is strongly recommended in all post-ICI resections to confirm R0 status, as the consequences of an inadvertently positive margin are amplified in the post-ICI context where re-resection is technically prohibitive [30]. Communication with pathology in advance of the case, alerting them to the expected gross specimen appearance and requesting prospective examination of suspicious areas, optimizes the yield of intraoperative consultation.

## 6. Emerging Platform Integration: RAB + RATS and AI-Assisted Navigation

### 6.1. Single-Anesthetic RAB + RATS: Diagnosis, Staging, and Resection in One Setting

One of the most compelling emerging paradigms in thoracic oncology is the integration of robotic-assisted bronchoscopy (RAB) with RATS into a single anesthetic event that encompasses diagnosis, mediastinal staging, and definitive resection [31]. This approach is particularly relevant in the post-ICI context, where the anatomical distortion described in preceding sections makes preoperative biopsy and staging increasingly important for operative planning. RAB platforms, including the Monarch (Auris Health), Ion (Intuitive Surgical), and Galaxy (Noah Medical) systems, employ shape-sensing catheter technology, electromagnetic navigation, and, in the case of Galaxy, tool-in-lesion (TiL) confirmation to navigate to peripheral pulmonary lesions with diagnostic yields exceeding 90% in prospective multicenter studies [32].

The single-anesthetic RAB + RATS workflow offers several specific advantages in the post-ICI patient. First, it eliminates the interval between biopsy and resection during which interim ICI administration might further remodel the operative field. Second, it allows real-time EBUS-guided mediastinal staging to confirm the pathological status of N2 nodes immediately before thoracoscopic resection, permitting intraoperative decision-making about the extent of surgery. Third, it avoids the cumulative sedation and pulmonary function burden of multiple separate procedures in a patient whose respiratory reserve may be compromised by ICI-related pneumonitis [31]. Logistically, this workflow requires operating room setup for both bronchoscopy (single-lumen intubation, fluoroscopy capability) and thoracoscopy (lung isolation, robotic docking) with a transition protocol between phases, ideally without changing patient position.

### 6.2. Single-Port (Uniportal) RATS: Current Evidence and Post-ICI Feasibility

Uniportal RATS, performed through a single working port with the da Vinci SP system or analogous single-incision robotic platforms, represents the frontier of minimally invasive thoracic surgery [33]. The SP system provides access through a single 2.5 cm port, with three articulating instruments and a flexible 3D camera emanating from a single cannula. Over 100 SP cases have been reported by Lee and colleagues, with acceptable safety profiles and a learning plateau observed at approximately 20–25 cases [5]. The advantages of uniportal RATS (minimal chest wall trauma, potential for reduced pain, single incision) must be weighed against its higher technical complexity, particularly in cases requiring extensive lymphadenectomy or management of vascular complications.

The feasibility of SP RATS specifically in post-ICI patients has not been formally studied, but the platform’s design characteristics present both advantages and potential limitations in this context. The enhanced dexterity and articulation of the SP instruments may facilitate dissection in the narrow, adhesion-filled spaces of the post-ICI hilum. However, the reduced number of working instruments compared to multiport configurations may limit the surgeon’s ability to simultaneously provide exposure, dissect, and control bleeding in the event of an intraoperative vascular injury. Until dedicated series specifically addressing SP RATS in post-ICI patients are published, multiport robotic approaches should be considered the standard for this challenging population at experienced centers.

### 6.3. AI-Assisted Augmented Reality and 3D Reconstruction for Distorted Anatomy

The application of artificial intelligence to preoperative planning and intraoperative navigation is advancing rapidly and holds particular promise in the post-ICI context, where conventional anatomical landmarks are unreliable [34]. AI-driven 3D reconstruction of preoperative CT imaging is commercially available through platforms integrated with robotic surgical systems and can generate patient-specific vascular maps that identify the precise origin, course, and branching pattern of pulmonary arterial and venous structures as they relate to the tumor and its post-ICI fibrotic envelope [34]. These reconstructions, displayed on the surgical console during the procedure, allow the operating surgeon to anticipate vascular anatomy before entering a tissue plane rather than discovering it during dissection.

Augmented reality (AR) overlay provides the superimposition of 3D vascular models onto the live endoscopic image and is currently at the clinical validation stage for thoracic surgery [35]. Early feasibility reports describe acceptable registration accuracy in non-ICI-treated patients. In the post-ICI context, where tissue deformation from fibrosis and adhesiolysis may progressively de-register the static preoperative model from the real-time surgical field, continuous model updating during the procedure will be essential. Automated performance metrics are algorithms that analyze instrument movement patterns, tissue interaction forces, and operative efficiency and are being developed as both quality assurance tools and training aids [35]. Their application to the post-ICI RATS context could enable objective identification of surgeons operating near the limits of safe dissection, triggering real-time advisory feedback.

### 6.4. Automated Performance Metrics and Surgical Quality Assessment

Beyond navigation, AI-based tools for intraoperative performance assessment are an emerging frontier in surgical quality improvement with direct relevance to complex post-ICI cases [36]. Machine learning models trained on video recordings of robotic thoracic procedures can identify operative phases, quantify instrument motion economy, and flag deviations from safe dissection trajectories. These tools are being evaluated not only as prospective safety aids but also as objective metrics for credentialing and training in robotic surgery programs [36]. For the post-ICI context specifically, where the operative complexity exceeds that of standard robotic lobectomy, such metrics may eventually inform minimum volume thresholds and institutional credentialing standards for post-ICI RATS, a regulatory question that remains entirely unaddressed.

## 7. Training, Credentialing, and Program Development

### 7.1. The Learning Curve for RATS in the Post-ICI Context

The learning curve for robotic lobectomy in upfront resection populations has been characterized in numerous single-institution series, with most estimates placing the plateau of proficiency at 20–40 cases for operative efficiency outcomes and 50–70 cases for complication rate stabilization [37]. The SORTS UK national survey has been the most comprehensive assessment of robotic thoracic surgery training availability in the United Kingdom and has demonstrated low national exposure to RATS among trainees and highlighted the need for structured curricula and simulation-based training pathways [5]. These findings reflect a global reality: RATS after ICI therapy demands a level of operative experience that exceeds the learning curve for standard robotic lobectomy, and surgeons who are themselves still on the upslope of that curve should not be undertaking post-ICI cases independently.

The post-ICI learning curve has not been formally quantified, and this is an important gap in the literature. Given the substantially higher technical burden documented in the available series, it is reasonable to hypothesize that the proficiency threshold for post-ICI RATS is considerably higher than for upfront robotic lobectomy. A pragmatic approach adopted by experienced centers is to require that surgeons demonstrate mastery of at least 50–100 upfront robotic lobectomies before independently undertaking post-ICI cases, and to perform initial post-ICI cases with an experienced robotic thoracic surgeon as a mentoring second console operator.

### 7.2. Simulation, Mentorship Models, and Credentialing Considerations

Box trainers, virtual reality simulators, and cadaveric model programs have all been deployed for robotic thoracic surgery training, though high-fidelity simulators that replicate the tissue characteristics of post-ICI anatomy (dense adhesions, friable vessels) do not yet exist [37]. Cadaveric training programs using formalin-fixed or fresh-tissue specimens with artificially created pleural adhesions represent the current best approximation, though the biomechanical properties of post-ICI tissue are distinct from any cadaveric preparation. The development of purpose-built simulation environments for post-ICI thoracic surgery should be a priority for the robotic thoracic surgery community.

Institutional credentialing for post-ICI RATS should be considered separately from credentialing for upfront robotic lobectomy, given the distinct technical demands. Minimum volume thresholds, mandatory proctorship for initial cases, and regular institutional morbidity and mortality review with case-specific analysis of post-ICI outcomes are reasonable quality assurance measures [37]. Centralization of post-ICI RATS to designated high-volume centers, similar to the esophagectomy volume-outcome relationship established in the thoracic surgery literature, may represent the safest model for patient care in the short to medium term.

## 8. Conclusions and Future Directions

The convergence of ICI-based neoadjuvant therapy and RATS represents one of the defining technical challenges and opportunities of contemporary thoracic oncologic surgery. The evidence reviewed here supports a coherent and clinically actionable synthesis. First, ICI therapy produces a distinct and predictable biological substrate in the operative field: hilar fibrosis, vascular friability, reactive lymphadenopathy, and tissue plane obliteration that do not correlate linearly with depth of pathological tumor response, as illustrated in Figure 1. Second, in retrospective series from high-volume centers, RATS appears to offer a technically favorable platform for navigating this environment, with 3D visualization, wristed instrument articulation, and tremor filtration translating into preserved minimally invasive completion rates in the post-ICI context; these advantages are most fully realized at experienced centers and their generalizability to lower-volume settings requires prospective evaluation. Third, emerging platform integrations (RAB + RATS in a single anesthetic event, uniportal RATS, and AI-assisted anatomical navigation) have the potential to further extend the frontier of what is achievable minimally invasively in post-ICI patients. The integrated decision pathway summarized in Figure 2 provides a practical framework for translating these principles into operative practice.

The limitations of the current evidence base must be acknowledged without equivocation. The available literature is dominated by retrospective single-center series, often from institutions with the highest global robotic thoracic volumes, limiting generalizability. Standardized intraoperative outcome definitions—particularly for conversion, technical difficulty grading, and lymph node assessment—are absent, precluding meaningful cross-study comparison. Long-term oncologic follow-up is limited or absent. Patient-reported outcomes, quality of life, and cost-effectiveness analyses are entirely lacking in the post-ICI RATS context.

The research agenda for the coming decade should prioritize the following: (1) prospective multicenter registries with standardized intraoperative data collection specifically for RATS after ICI therapy; (2) randomized or propensity-matched comparisons of RATS versus VATS versus open surgery in post-ICI patients with a priori defined endpoints including conversion rate, lymph node yield, R0 rate, and long-term survival; (3) development and validation of preoperative prediction models (incorporating clinical, radiologic, and biological variables) for intraoperative technical difficulty after ICI therapy; (4) formal learning curve analysis for post-ICI RATS and evidence-based credentialing standards; and (5) prospective evaluation of the single-anesthetic RAB + RATS paradigm in terms of oncologic completeness, operative efficiency, and patient-centered outcomes. Until such evidence matures, the surgeon operating in the post-ICI era must combine technical mastery, meticulous preoperative planning, a prepared and experienced team, and a willingness to convert early and without hesitation when patient safety demands it.

## Figures and Tables

**Table 2:** Intraoperative challenges in post-ICI robotic-assisted thoracic surgery (RATS) and corresponding RATS-specific technical adaptations.

Challenge	ICI-Related Mechanism	RATS-Specific Adaptation	Rationale
Obliterated pleural space/dense adhesions	Paratumoral inflammation; pleural fibrosis from ICI activation	Diagnostic thoracoscopy before docking; adhesiolysis under direct vision; adjust port sites to adhesion map	Prevents blind trocar injury; identifies safe access windows before robot is committed
Fused interlobar fissures	TGF-β-mediated fibroblast activation obliterates fissure planes	Fissure-last (fissureless) technique; PA and bronchus secured before fissure division	Avoids entry into fissural vascular plexus; robotic wristed instruments navigate tight planes without fulcrum
Periadventitial PA fibrosis	IFN-γ-driven periadventitial inflammation; vascular wall fragility	Cold sharp dissection with robotic scissors along adventitia; clip-based haemostasis; avoid energy near PA	Thermal spread from energy devices risks dehiscence of inflamed vessel wall
Enlarged calcified reactive nodes	T-cell infiltration, germinal centre formation, reactive lymphadenopathy with calcification	Node-by-node capsular dissection; 10× magnified 3D visualisation; robotic tips navigate narrow corridors	Tremor filtration and wristed articulation allow precise capsular plane identification impossible with VATS
pCR-related surgical complexity	Maximal immune activation → maximal fibrotic response despite absent tumour	Pre-case alert to team; low conversion threshold; intraoperative frozen section of all margins	pCR does not predict easy surgery; radiologic CR may coincide with densest hilar fibrosis
Intraoperative vascular haemorrhage	Friable inflamed vessel wall; haemostatic compromise from periadventitial fibrosis	Undocking protocol rehearsed pre-case (<60 s); bedside assistant applies tamponade; open tray on field	Console surgeon distant from field; bedside assistant independence is critical for emergency management
Margin assessment in pCR specimens	Fibrohyaline replacement of tumour bed grossly indistinguishable from normal parenchyma	Mandatory intraoperative frozen section of bronchial and vascular margins; pathology pre-briefed	Visual R0 assessment unreliable in pCR; re-resection technically prohibitive post-ICI

ICI, immune checkpoint inhibitor; IFN-γ, interferon-gamma; PA, pulmonary artery; pCR, pathological complete response; RATS, robotic-assisted thoracic surgery; TGF-β, transforming growth factor-beta; VATS, video-assisted thoracoscopic surgery.

## Data Availability

No new data was generated in this review article.
